# Urban air particulate matter induces mitochondrial dysfunction in human olfactory mucosal cells

**DOI:** 10.1186/s12989-020-00352-4

**Published:** 2020-06-01

**Authors:** Sweelin Chew, Riikka Lampinen, Liudmila Saveleva, Paula Korhonen, Nikita Mikhailov, Alexandra Grubman, Jose M. Polo, Trevor Wilson, Mika Komppula, Teemu Rönkkö, Cheng Gu, Alan Mackay-Sim, Tarja Malm, Anthony R. White, Pasi Jalava, Katja M. Kanninen

**Affiliations:** 1 A.I. Virtanen Institute for Molecular Sciences, University of Eastern Finland, Kuopio, Finland; 2 Department of Anatomy and Developmental Biology, Monash University, Wellington Road, Clayton, Victoria Australia; 3 Development and Stem Cells Program, Monash Biomedicine Discovery Institute, Wellington Road, Clayton, Victoria Australia; 4 Australian Regenerative Medicine Institute, Monash University, Wellington Road, Clayton, Victoria Australia; 5 Hudson Institute of Medical Research, Clayton, VIC 3168 Australia; 6 Finnish Meteorological Institute, Kuopio, Finland; 7 Inhalation Toxicology Laboratory, Department of Environmental and Biological Sciences, University of Eastern Finland, Kuopio, Finland; 8 School of the Environment, Nanjing University, Nanjing, China; 9 Griffith Institute for Drug Discovery, Griffith University, Nathan, QLD 4111 Australia; 10 QIMR Berghofer Medical Research Institute, Herston, QLD 4006 Australia

**Keywords:** Mitochondria, olfactory system, particulate matter, Air pollution, *NPTX1*, Inflammation, oxidative stress

## Abstract

**Background:**

The adverse effects of air pollutants including particulate matter (PM) on the central nervous system is increasingly reported by epidemiological, animal and post-mortem studies in the last decade. Oxidative stress and inflammation are key consequences of exposure to PM although little is known of the exact mechanism. The association of PM exposure with deteriorating brain health is speculated to be driven by PM entry via the olfactory system. How air pollutants affect this key entry site remains elusive. In this study, we investigated effects of urban size-segregated PM on a novel cellular model: primary human olfactory mucosal (hOM) cells.

**Results:**

Metabolic activity was reduced following 24-h exposure to PM without evident signs of toxicity. Results from cytometric bead array suggested a mild inflammatory response to PM exposure. We observed increased oxidative stress and caspase-3/7 activity as well as perturbed mitochondrial membrane potential in PM-exposed cells. Mitochondrial dysfunction was further verified by a decrease in mitochondria-dependent respiration. Transient suppression of the mitochondria-targeted gene, neuronal pentraxin 1 (*NPTX1*), was carried out, after being identified to be up-regulated in PM_2.5–1_ treated cells via RNA sequencing. Suppression of *NPTX1* in cells exposed to PM did not restore mitochondrial defects resulting from PM exposure. In contrast, PM-induced adverse effects were magnified in the absence of NPTX1*,* indicating a critical role of this protein in protection against PM effects in hOM cells.

**Conclusion:**

Key mitochondrial functions were perturbed by urban PM exposure in a physiologically relevant cellular model via a mechanism involving *NPTX1*. In addition, inflammatory response and early signs of apoptosis accompanied mitochondrial dysfunction during exposure to PM. Findings from this study contribute to increased understanding of harmful PM effects on human health and may provide information to support mitigation strategies targeted at air pollution.

## Introduction

The World Health Organization reported that 90% of the world population breathe polluted air [1]. The detrimental effect of urban air pollutants including particulate matter (PM) on human health has long been subjected to intense investigation. The toxic effects of PM smaller than 2.5 μm in diameter (PM_2.5_) on human lung epithelial and immune cell lines are well-characterized [2, 3]. These studies have demonstrated that exposure to PM triggers oxidative stress and inflammation. Specifically, Lavrich et al. reported PM induced functional perturbation in human lung cell mitochondria, which play a critical role in energy metabolism and oxidative stress [4]. However, the exact mechanisms through which the PM drive oxidative stress or inflammation remain largely unknown.

More recent evidence suggests that in addition to peripheral effects, PM exposure adversely affect the central nervous system. In fact, epidemiological studies have demonstrated that exposure to urban air pollutants is associated with neurodegenerative disorders including dementia and cognitive impairment [5, 6]. Since the discovery of potential adverse brain effects of PM exposure, the entry route of pollutants to the brain has been the subject of intense investigation. In addition to entering the brain from the bloodstream [7], it has been shown that translocation of small inhaled pollutant particles to the brain can occur via the olfactory system [8].

The olfactory system transmits smells to the olfactory bulb via the olfactory nerve. Impaired olfactory function is a common, early sign of neurodegenerative diseases [9], yet its role in these disorders is not fully understood. The olfactory mucosa (hOM), which is located on the upper part of the nasal cavity closest to the cribriform plate, acts as the first line of defence against inhaled agents, including ambient particles [10]. The hOM is a well-characterized tissue which harbors horizontal basal cells, a small population of multipotent stem cells that self-renew and give rise to globose basal cells, sensory neurons, and sustentacular cells [11, 12]. Animal studies have shown that both nasal and olfactory epithelial barriers are disrupted upon exposure to concentrated urban PM [13, 14]. Similarly, nanoparticle deposition has been demonstrated in the human OM, a key entry site of ambient pollutant particles to the brain [15].

Here we present a novel, highly physiologically relevant cellular model of the hOM as a tool to investigate mechanisms and consequences of air pollutant exposure. We postulate that PM exposure at the hOM targets the mitochondria, thereby inducing oxidative stress and inflammation. We report that PM exposure of hOM cells results in critical impairment of mitochondrial function, that manifests as increased oxidative stress. We also reveal novel cellular targets of PM exposure and identify NPTX1 as a key mediator that initiates mitochondrial dysfunction and subsequent apoptosis.

## Methods

### Reagents

All reagents for cell culture were purchased from Thermo Fisher Scientific (Waltham, USA) unless otherwise stated.

### Cell culture

Primary hOM cultures from four healthy male individuals (aged 54–68 years) were established as described [16] with ethical approval from Griffith University under the project number ESK/01/11/HREC. Further information on the cell lines are presented in Table S1 in Supplementary Information. Briefly, olfactory mucosa were collected under local anesthesia from healthy volunteers by biopsy of the nasal septum approximately 1 cm from the roof of the nasal cavity, as reported [17, 18]. Once hOM cultures were established, they were maintained for experiments in growth medium containing DMEM/F12 (#11320033) supplemented with 10% heat-inactivated FBS (#10270106) and 1x Penicillin-Streptomycin (#15140122), at 37 °C, 5% CO_2_. Cells between primary passage 6 and 9 were analyzed. For gene expression analyses, cells were also cultured in neurosphere induction media (NIM) containing DMEM/F12, 1x Penicillin-Streptomycin (#15140122), 1x ITS-G supplement (#41400045), 50 ng/ml recombinant human EGF (Peprotech Nordic, Stockholm, Sweden) and 25 ng/ml recombinant human basic FGF (Peprotech) as well as neuronal media (NM), containing Neurobasal Medium (#21103049), 1x B27 supplement (#17504044), 1x Penicillin-Streptomycin (#15140122) and 25 mM L-glutamine (#25030081).

### PM preparation and exposure

Urban size-segregated PM_1–0.2_, PM_2.5–1_ and PM_10–2.5_ were collected from Nanjing, China with a high volume cascade impactor and reconstituted as described [3]. Detailed information on the collection site, meteorology, chemistry and protocols in Table S2 in Supplementary Information and are described [3, 19]. Despite high concentrations of PM in the sampling location, the mass of the ultrafine particles was relatively low. The vehicle control was prepared by mixing sterile water (Baxter, Ontario, Canada) with 3% v/v sterile DMSO (Sigma-Aldrich, St. Louis, USA) and added to the medium at 1:100 dilution. hOM cells were then exposed to culture medium containing vehicle or PM at 50 μg/ml for 4 or 24 h at 37 °C, 5% CO_2_.

### Measurement of metabolic activity, toxicity and apoptosis

After a 24-h exposure to PM, the culture media was removed for quantification of lactate dehydrogenase (LDH) release with Pierce LDH Cytotoxicity kit (#88954) and performed according to the recommended protocol. Absorbances were read at 490 nm and 650 nm using Wallac Victor 1420 plate reader (Perkin Elmer, Waltham, USA). Lysed cells were used as a reference for the cytotoxicity assay. To assess metabolic activity, the PM-exposed cells were incubated in medium containing 1.2 mM thiazolyl blue tetrazolium bromide (Sigma-Aldrich, St. Louis, USA) at 37 °C for 2 h. The culture media was removed and DMSO (Sigma-Aldrich, St. Louis, USA) was added to the cells to solubilize the salt. Absorbance was then read at 595 nm using Wallac Victor 1420 plate reader. Absorbance was normalized to the vehicle and lysed cells were used as a negative control. Apoptosis was assessed with CytoFLEX S Flow Cytometer (Beckman Coulter Life Sciences, Brea, USA) using CellEvent caspase-3/7 Green ReadyProbes™ Reagent (#R37111), according to the manufacturer’s protocol.

### RNA isolation and quantitative real-time PCR (qPCR)

Total RNA and DNA was extracted using TRI reagent (Sigma Aldrich, St. Louis, USA), according to the commercial protocol. The concentration and purity of RNA samples were determined using NanoDrop 1000. cDNA was synthesized from 1 μg RNA with the High Capacity Reverse Transcription kit (#4368814). The relative expression levels of mRNAs encoding the selected genes were analyzed in triplicates and measured according to the manufacturer protocols using StepOnePlus™ Real-Time PCR System (#4376600). qPCR was carried out on 10 ng cDNA or genomic DNA using Maxima Probe/ROX qPCR Master Mix (#K0231). Taqman gene expression assays used in this study are listed in Table S3 in Supplementary Information. Relative mRNA expression was calculated with the 2 − ∆∆Ct method where Ct is the threshold cycle number and results presented as values in relation to the control conditions. Ct values up to 30 are considered expressed and fold changes are normalized to h*GAPDH* and to the vehicle.

### Flow cytometry

Reactive oxygen species (ROS) were quantified using H2DCFHA (#D399), CellROX Deep Red (#C10422) and MitoSOX (#M36008). After 24-h exposure to PM, the cells were incubated in growth medium containing 5 μM ROS indicators for 30 min at 37 °C. The cells were then resuspended in PBS containing 1x SYTOX® Blue (#S34857), 1% inactivated FBS v/v, 2 mM EDTA (Sigma-Merck) and 0.05% sodium azide w/v. All samples were immediately analyzed using the CytoFLEX S Flow Cytometer (Beckman Coulter). STYOX® blue, H2DCFHA, CellROX Deep Red and MitoSOX were read at emission wavelengths 450 nm, 525 nm, 660 nm and 580 nm respectively. Signal intensity of the positive population in all channels was gated at 10^4^ AU for the cell area. Signal in 10,000 live cells were acquired and the average signal intensities of the live cell population were presented.

### Cytokine secretion measurement

To assess the inflammatory response in the hOM cultures, cells were incubated in media containing vehicle or PM, supplemented with IFNɣ (PeproTech Nordic, Stockholm, Sweden) at 7.5 ng/ml and TNFα (PeproTech Nordic, Stockholm, Sweden) at 5 ng/ml. After a 24-h incubation at 37 °C, 20 μl of media was collected to quantify secreted levels of IL6, IL8, RANTES, GM-CSF and MCP1 using the Cytometric Bead Array (CBA) Human kit (BD Biosciences, California, USA). Data was acquired using CytoFLEX S (Beckman Coulter) and analyzed with FCAP Array™ v2.0.2 software (Soft flow Inc., Minnesota, USA).

### Live-cell analysis of mitochondrial membrane potential

All solutions used for imaging were diluted to final concentrations from stock solutions with basic salt solution (BSS) containing (in mM): 152 NaCl, 2.5 KCl, 10 HEPES, 10 glucose, 2 CaCl_2_, 1 MgCl_2_ (pH adjusted to 7.4). Prior to experiments, cells were loaded with 5 μM Rho123 (Molecular probes, 5 mM stock solution in 99% ethanol) for 30 min at 37 °C. Then cells were transferred to TILL Photonics imaging system (TILL Photonics GmbH, Munich, Germany) where they were continuously perfused with BSS. The setup was equipped with fast perfusion system (Rapid Solution Changer RSC-200, BioLogic Science Instruments, Grenoble, France), which allowed fast exchange between applying solutions (exchange time ~ 30 ms). Cells were imaged with Olympus IX-70 (Tokyo, Japan) microscope using 20× objective and 495 nm excitation light. Images were collected using CCD camera (SensiCam, PCO imaging, Kelheim, Germany) with sampling frequency set to 1 frame per second. Cells were characterized by the maximum fluorescence of their responses to two-minute application of 4 μM FCCP (Abcam, 20 mM stock solution in DMSO). To obtain baseline fluorescence, prior to application of FCCP cells were perfused for one minute with BSS contained same concentration (0.02% v/v) of DMSO as FCCP solution. Regions of interest were chosen in nuclei of cells and maximum response to FCCP were normalized to the baseline. In total, responses of 160 cells were collected for statistics for each treatment (four different cell lines as different biological replicates, four technical replicates for each cell line, ten cells from each technical replicate).

### Mitochondrial respiration assay

Mitochondrial metabolism was analyzed with the Seahorse XF24 analyzer and Mitostress Test according to the manufacturer’s instructions (Agilent Technologies). hOM cells were seeded at a density of 50,000 cells/well on a XF24 cell culture microplate (Seahorse Bioscience). The next day, hOM cells were exposed to 50 μg/ml PM for 24 h at 37 °C. Working concentrations of oligomycin, FCCP, rotenone and antimycin A (Sigma) used to carry out the Mitostress Test were 1.264 μM, 1 μM, 0.5 μM and 0.5 μM respectively. The oxygen consumption rates were normalized to protein content per well, measured using the Pierce BCA Protein Assay Kit (#23225), according to the commercial protocol. The results were analyzed with Wave Desktop 2.4 (Agilent Technologies, Santa Clara, CA, USA) according to the manufacturer’s instructions. The parameters of mitochondrial function, including basal respiration, ATP production, proton leak, spare respiratory capacity and non-mitochondrial respiration were calculated from oxygen consumption rate (OCR) for basal and maximal energy demands. Basal respiration was derived by subtracting non-mitochondrial respiration from baseline OCR measured before addition of assay test compounds. The ATP-linked respiration was calculated by subtracting the OCR after addition of oligomycin, a complex V inhibitor, from baseline cellular OCR. The proton leak was derived by subtracting non-mitochondrial respiration from the oligomycin rate. Maximal respiratory capacity was calculated by subtracting non-mitochondrial respiration from the FCCP rate, since FCCP is a protonophore that collapses the inner membrane gradient allowing the electron transport chain to function at its maximal rate. Non-mitochondrial respiration was measured directly from OCR after addition of last assay rest compound mix, antimycin A and rotenone, which are inhibitors of complex III and I and shut down electron transport chain function. Mitochondrial reserve capacity was calculated by subtracting basal respiration from maximal respiratory capacity.

### Quantification of ATP

Following a 24-h treatment with PM, media was removed from cells and the cells were lysed for ATP levels measurement using the ATPLite Luminescence Assay System (Perkin Elmer, Waltham, MA, USA), according to the manufacturer’s instructions. Luminescence was read with the Wallac Victor 1420 Multiplate reader (Perkin Elmer, Waltham, MA, USA).

### Transcriptomic analysis

Total RNA was extracted from 150,000 cells exposed to PM_2.5–1_ and vehicle using RNeasy Mini Kit (Qiagen, Hilden, Germany). The quality of the RNA was analyzed on the 2100 Bioanalyzer with the RNA 6000 Pico Kit (both from Agilent, California, USA), according to the recommended protocol. Residual Genomic DNA was removed using RNAse-free DNase I (#EN0521) and re-purified using Agencourt RNAClean XP beads (Beckman Coulter). First strand synthesis was carried out using a custom dT primer which adds an 8 bp sample index and 10 bp unique molecular identifier to the poly-A end of transcripts. The tagged cDNA is pooled and amplified using a template switching oligonucleotide and the Illumina P5 and P7 sequences added by PCR and Nextera transposase respectively. Sequencing was carried out using paired end reads on a NextSeq HO v2.5 run with a custom R1 primer to sequence the Index/UMI (18 bp) and 70 bp R2 to sequence the cDNA. Each sample was sequenced at a depth of approximately 17 million reads and the data was generated as FASTQ files.

### Demultiplexing and mapping

Sequencing reads were processed using an in house pipeline consisting of sabre tools (https://github.com/serine/sabre) and RNAsik [20]. Samples were demultiplexed with a fork of sabre tools with the commands below. After demultiplexing raw data was processed with RNAsik pipeline to generate QC metrics, including percentage of reads mapped and assigned to the reference genome and duplication rates, and raw read counts for differential expression analysis. Demultiplexed UMI tagged sequencing reads were aligned to the human genome (Ensembl GRCh38 primary assembly) using RNAsik. Read deduplication based on UMIs was performed with Je markdupes in RNAsik and transcript read counts calculated with featureCounts [21].

### GO enrichment analysis

Enrichment of up- or down-regulated differentially expressed genes (DEGs) at false discovery rate (FDR) cut-off of 0.05 was carried out using Gene Ontology (GO) (http://geneontology.org/) Enrichment Analysis [22, 23]. GO terms including biological process and molecular functions with FDR ≤0.05 were enriched.

### Transient *NPTX1* knock-down

To achieve at least 75% knock down in *NPTX1* transcript levels, cells at 80% confluence were transfected with a pool of 3 *NPTX1*-targeted dsiRNA from the TriFECTa® RNAi kit (iDT, Coralville, US) to a final concentration of 10 nM. Transfection was carried out in optiMEM (#31985070) using Metafectene SI+ (Biontex, Munich, Germany) and performed according to manufacturer’s recommendation. After 48 h incubation, the cells were exposed to PM for 24 h before downstream assays.

### Statistical analysis

Cell culture experiments were carried out in triplicates in four biological replicates. Data is reported as mean ± SD and the experiments were carried out in four cell lines, each with at least three technical replicates unless otherwise indicated. *p-*values below 0.05 were considered statistically significant. Statistical analyses were performed with GraphPad Prism 5.03 (GraphPad Software, San Diego, CA, USA) using repeated measures ANOVA for comparison of multiple donors and ANOVA for single donors, followed by Bonferroni post hoc tests to compare means with the vehicle, unless stated otherwise assuming homoscedasticity and normality of variables. Statistically significant outliers, calculated via Grubb’s tests (GraphPad), were excluded from analysis.

## Results

### hOM cultures display a heterogenous population of cells

**Fig. 1:**
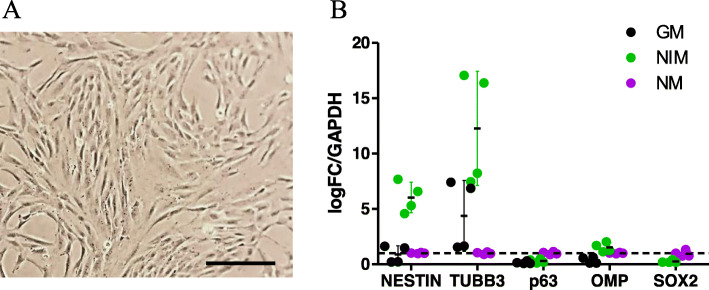
Morphology and gene expression profile of hOM cultures. **a** Representative phase-contrast image of hOM cells cultured as a monolayer. Scale bar = 100 μm. **b**. Comparison of DEGs of a representative hOM cell line cultured in growth medium (GM), neurosphere induction medium (NIM) and neuronal medium (NM). Relative transcript levels of several cell markers including nestin, *SOX2* and *TP63* are specific to basal stem cells. *SOX2*, *PAX6* and *TP63* are specific to cells committed to sustentacular cell fate. * TUBB3* and *OMP* are specific to cells committed to olfactory sensory neuronal cell fate. Transcript levels are normalised to GM with *hGAPDH* as a reference gene, *n* = six wells/group

hOM cultures were obtained from nasal biopsies as described earlier [16]. Cell morphology and gene expression profiles were used to characterize the composition of the newly established primary hOM cultures. When cultured in growth medium, the cells displayed a morphology resembling epithelial cells (Fig. 1a) and were observed to have a doubling time of approximately 24 h. Subject-to-subject variation in growth rate was apparent only in cells sub-cultured beyond primary passage 10. qPCR was used to decipher the gene expression levels of biomarkers for basal cells, sensory neurons, and sustentacular cells (Fig. 1b). The hOM cultures express certain genes associated with cell types reported to be located on the OM; basal cells (*nestin, SOX2* and *TP63*), olfactory sensory receptor neurons (*TUBB3, OMP*) and sustentacular cells (*SOX2*). The expression levels of mature neuronal markers, *NEUROG1, ASCL1, DCX* and glial marker, *S100ß* were below detection limits (data not shown). A comparison of the gene expression levels of hOM cells cultured in growth media, neurosphere-induction medium and neuronal medium revealed only slight differences. Nestin and TUBB3 were the only marker whose expression levels were altered in different culture conditions, being highest when cultured in the neurosphere-induction medium. Taken together, these results demonstrate that the hOM cultures express markers of cell types in the hOM in vivo, thus providing evidence for its physiological relevance and suitability in studies of air pollution effects. Given the observed minor alterations in gene expression profiles due to different culture media compositions, we chose to carry out all subsequent experiments in the growth media to minimize growth factor effects on the cell metabolism.

### Exposure to PM perturbs cellular metabolism in hOM cultures

**Fig. 2:**
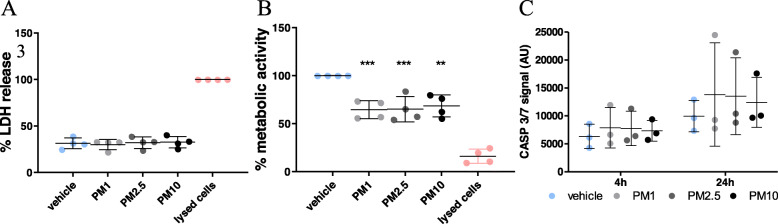
Effects of PM on cytotoxicity and cellular metabolism in hOM cultures. **a** hOM cells exposed to PM_1–0.2_, PM_2.5–1_ or PM_10–2.5_ for 24 h, after which cell death was measured by the LDH release assay. Results were normalised by lysed cell control, n = four donors. Metabolic activity was measured by the MTT reduction assay of hOM cultures. Results were normalised to the vehicle, n = four donors. ** *p* < 0.01, *** *p* < 0.001. **c** Caspase-3/7 signal intensity in live single cells of a representative culture exposed to all three PM, n = three experimental replicates

Having validated the physiological relevance of the hOM cultures, we then exposed the cultures to urban PM_1–0.2_, PM_2.5–1_ and PM_10–2.5_ at a concentration of 50 μg/ml. Initial metabolic activity and cytotoxicity assessment were carried out assessing PM concentrations ranging from 6.5 μg/ml to 100 μg/ml (see Figure S1). At 50 μg/ml, reduced metabolic activity in cells can be observed without overt cytotoxicity, allowing for some recapitulation of cellular damage in chronic exposure to PM in vivo [3, 19]. A 24-h incubation with the different PM size classes did not result in differences in LDH release into the culture medium (Fig. 2a). LDH release in the vehicle (31.41% ± 2.91%), PM_1–0.2_ (29.97% ± 2.71%), PM_2.5–1_ (32.08% ± 3.11%) and PM_10–2.5_ (32.64% ± 3.09%) was shown to be non-cytotoxic, compared to the lysed cell positive control (100%). However, an approximate 30% reduction in metabolic activity measured via MTT reduction was observed following PM exposure when compared to the vehicle. This may suggest that the PM may affect key cellular metabolic functions (Fig. 2b). Metabolic activity dropped significantly after exposure to PM_1–0.2_ (64.51% ± 4.69%), PM_2.5–1_ (65.15% ± 13.2%) and PM_10–2.5_ (68.49% ± 5.74%) when compared to the vehicle. However, no significant difference was observed between PM size classes. Similarly, flow cytometric analysis revealed an increase in signal intensity for caspase-3/7 activity after PM exposure, compared to the vehicle (Fig. 2c). These observations suggest that, while not causing cell death, a 24-h PM exposure perturbs cellular metabolism in the hOM cultures.

### An inflammatory response is observed following hOM culture exposure to PM

**Fig. 3:**
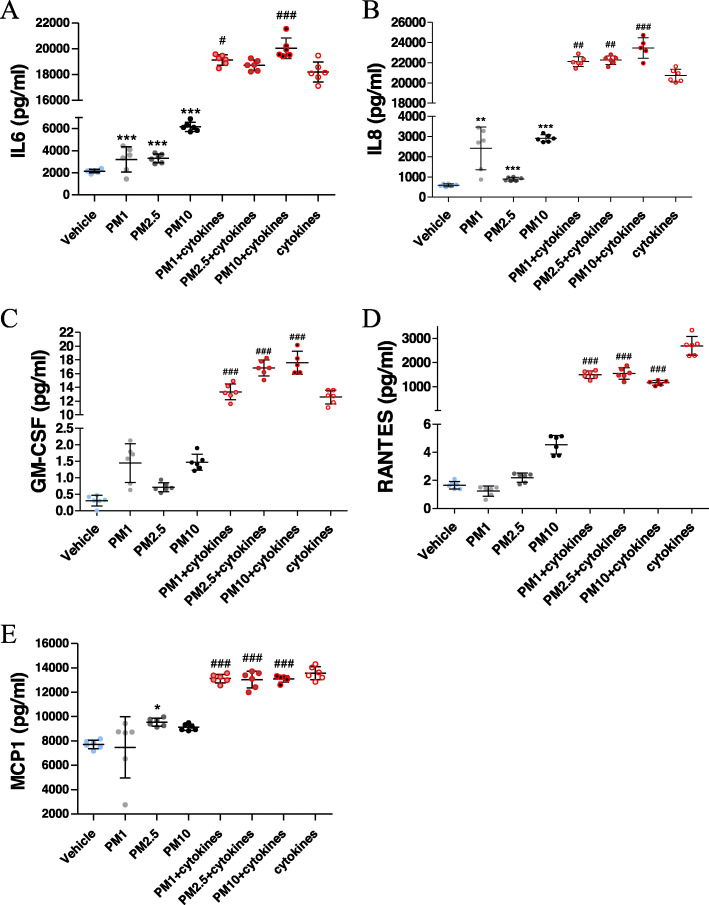
PM effects on the immune response in hOM cultures. Secreted inflammatory mediators were quantified from a representative cell line after 24-h h exposure by CBA. Following exposure to PM, the levels of of (**a**) IL-6, (**b**) IL-8, (**c**) GM-CSF, (**d**) RANTES and (**e**) MCP1 in hOM cultures were measured. After a 24 h co-treatment with IFN-ɣ/TNFα and PM_1–0.2_, PM_2.5–1_ or PM_10–2.5_, the levels of IL-6, IL-8, GM-CSF, RANTES and MCP were determined by CBA. *p < 0.05, ***p < 0.001, n = five wells/group and compared to the vehicle. #*p* < 0.05, ##*p* < 0.001, ###*p* < 0.0001, compared to cytokines

To decipher the cellular mechanisms via which PM exposure triggered deteriorating metabolism in the hOM cultures, we first assessed cytokine release, a key process of the cellular inflammatory response. The CBA was carried out on collected cell culture media to measure secreted levels of pro-inflammatory cytokines and chemokines after a 24-h exposure to PM. In addition, hOM cultures were stimulated by IFN-ɣ/TNFα as a positive control for cytokine secretion. Exposure to the different size classes of PM alone caused a slight elevation in the secretion of IL6, IL8, GM-CSF, RANTES and MCP-1 (Fig. 3a-e). When compared to PM_1–0.2_ and PM_2.5–1_, treatment with PM_10_ elicited the strongest secretion of cytokines, indicating a PM size-dependent pattern of cytokine/chemokine release.

To compare the magnitude of cytokine release from hOM cultures upon PM exposure to that of a substantial inflammatory insult, we next measured the cytokine levels in cells treated with the positive control IFN-ɣ/TNFα alone, or in combination with the PM. When compared to treatment with PM alone, the cytokine secretion was dramatically increased when hOM cultures were treated with IFN-ɣ/TNFα (Fig. 3a-e). A combination treatment of IFN-ɣ/TNFα with the three PM sizes revealed only slight additive effects. IFN-ɣ/TNFα together with PM_10_ only further augmented the secretion of IL-6 and GM-CSF at 20039 ± 791 pg/ml and 18 ± 2 pg/ml respectively when compared to IFN-ɣ/TNFα alone at 18202 ± 790 pg/ml and 13 ± 1 pg/ml respectively (Fig. 3a and c). GM-CSF levels were also further augmented in concomitant exposure to PM_2.5–1_ and IFN-ɣ/TNFα (Fig. 3c). Interestingly, regardless of size class, a combination treatment of all PM sizes with IFN-ɣ/TNFα reduced the secretion of RANTES over that of IFN-ɣ/TNFα alone (Fig. 3d). In contrast, slightly more MCP-1 was secreted by the hOM cells exposed to PM, when compared to the vehicle. Altogether, these results suggest that while exposure to PM results in low level cytokine secretion in hOM cultures that is at times PM size dependent, a robust induction of the inflammatory response is only observed upon treatment with the pro-inflammatory cytokines IFN-ɣ/TNFα. In addition, combining a pro-inflammatory insult to PM exposure also results in a differential IL-6, GM-CSF and RANTES secretion profile to that of IFN-ɣ/TNFα alone.

### PM treatment results in increased oxidative stress in hOM cultures

**Fig. 4:**
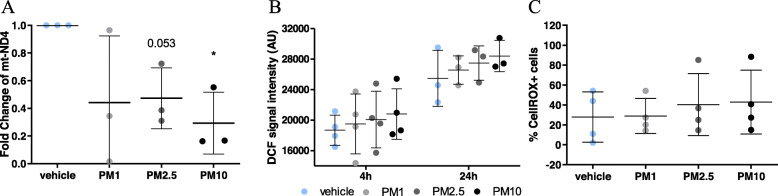
Elevated cellular ROS in hOM cultures after PM exposure. **a** Measurement of mt-ND4 content in hOM cultures after 24 h exposure to PM_1–0.2_, PM_2.5–1_ and PM_10–2.5_ . Data are normalized the vehicle treated cells and using the reference gene hGAPDH. **b** Cytoplasmic reactive oxygen intermediates were also detected via H2DCFHA (DCF) signal intensities examined in cells after 4 h and 24 h exposure in a representative cell line. **c** Cellular ROS levels quantified by CellROX reveals a slight increase in average CellROX-positive cells exposed to PM_2.5–1_ and PM_10–2.5_ . n = four donors

To ascertain the effects of PM on other cellular process often associated with exposure to air pollutants, we next focused on oxidative stress and mitochondrial DNA (mtDNA) content, which were monitored via flow cytometric analysis and qPCR respectively. Exposure to PM did not have a significant effect on mtDNA content, assessed by the mitochondrially encoded NADH:ubiquinone oxidoreductase core subunit 4 (mt-ND4). Next, measurement of intracellular ROS via both CellROX deep red and H2DCF-HA revealed a similar PM-induced increase in detected ROS levels following a 4-h exposure to all three PM size classes (Fig. 4b, c). Cellular ROS was detected in a slightly larger proportion of cells exposed to the PM (approximately 29 to 43% in three PM size classes), when compared to the vehicle (27.82% ± 25.24%). Moreover, cellular ROS levels were exacerbated following 24 h of exposure to PM and the vehicle (Fig. 4b). These findings imply the involvement of oxidative stress in cellular responses following PM exposure.

### Mitochondrial ROS production and membrane potential are disrupted upon PM exposure

**Fig. 5:**
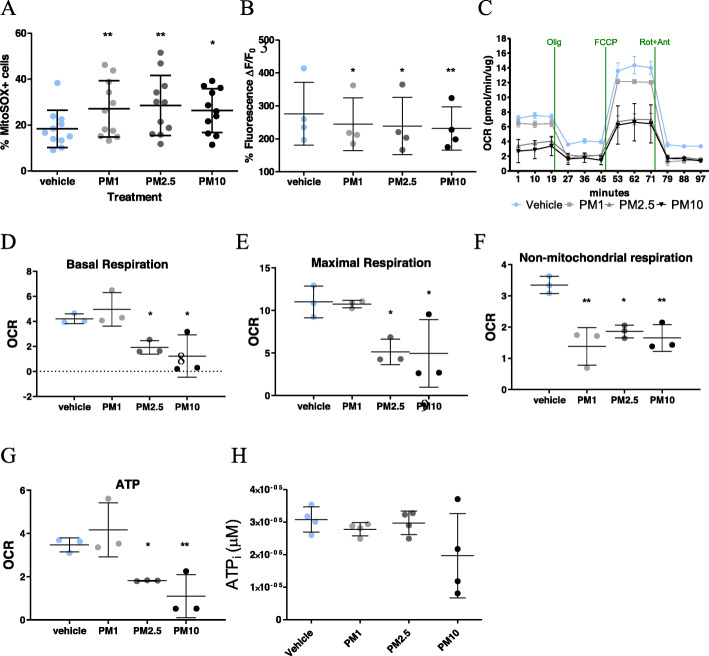
Exposure to PM increased mitochondrial ROS and altered key mitochondrial functions. **a** Mitochondrial ROS levels were quantified by MitoSOX labelling after 4 h exposure revealed a significant increase in cells exposed to PM_2.5–1_ and PM_10–2.5_ . **b** Significant loss of mitochondrial membrane potential was observed in all PM size classes. **c** Line plot displaying oxygen consumption rate (OCR) of hOM cultures exposed to PM and quantified in pmol/minute/μg after the addition of oligomycin (Olig), FCCP and Rotenone/Antimycin (Rot+Ant). **d** Basal respiration in cells exposed to PM_1–0.2_ (5.17 ± 0.59), PM_2.5–1_ (2.92 ± 1.03) and PM_10–2.5_ (2.32 ± 1.29), compared to the vehicle (4.44 ± 0.28). **e** Maximal respiration after exposure to PM_1–0.2_ (10.74 ± 0.26), PM_2.5–1_ (5.14 ± 0.86) and PM_10–2.5_ (4.96 ± 2.3), compared to the vehicle (11.01 ± 1.08). **f** Non-mitochondrial respiration after exposure to PM_1–0.2_ (1.38 ± 0.35), PM_2.5–1_ (1.86 ± 0.12) or PM_10–2.5_ (1.65 ± 0.25), compared to the vehicle (3.35 ± 0.16). **g** ATP production in hOM cells exposed for 24 h to PM_1–0.2_ (4.17 ± 0.72), PM_2.5–1_ (1.81 ± 0.01) or PM_10–2.5_ (1.1 ± 0.58), compared to the vehicle (3.47 ± 0.19). **h** Intracellular ATP levels reflected little variation in hOM cultures exposed to PM_1–0.2_ (0.28 μM ± 0.02 μM), PM_2.5–1_ (0.2 μM ± 0.04 μM) or PM_10–2.5_ (0.28 μM ± 0.13 μM) from vehicle control (0.31 μM ± 0.04 μM). The OCR was normalised by protein levels. * p < 0.05, **p < 0.01. n = four donors/group

To further examine the oxidative stress induced by PM exposure, we focused the studies on mitochondria, a major source of cellular ROS. PM effects on mitochondrial ROS levels and the mitochondrial membrane potential were examined via flow cytometry and Rhodamine123 imaging respectively. After a 4-h PM exposure, flow cytometric analysis of hOM cultures labelled with mitoSOX revealed a slightly larger population of mitoSOX-positive cells in cultures exposed to PM_2.5–1_ and PM_10–2.5_, when compared to the vehicle (Fig. 5a). Similarly, a slight loss of mitochondrial membrane potential after exposure to all three PM size classes compared to the vehicle was observed. Again, a PM-dependent reduction in membrane potential was evident (Fig. 5b). These results warranted a thorough investigation of PM effects in key mitochondrial functions in living hOM cultures.

### PM exposure impairs key mitochondrial functions in hOM cultures

In order to test the hypothesis that PM exposure impaired cellular respiration, PM effects on mitochondrial function in live cells were measured in real time using the Seahorse Mitostress assay. A 24-h exposure to PM_1–0.2_ did not appear to affect mitochondrial parameters as much as PM_2.5–1_ and PM_10–2.5_, as seen in Fig. 5c. PM_2.5–1_ and PM_10–2.5_ significantly reduced oxygen consumption rates (OCR) by two-fold in basal respiration (Fig. 5d). Maximal respiratory capacity also appeared reduced upon PM exposure (Fig. 5e). The OCR that is associated with non-mitochondrial respiration was dramatically reduced by exposure to all size classes of PM (Fig. 5f). The Mitostress test also revealed a significant reduction in ATP production (Fig. 5g) that did not quite reach statistical significance when intracellular ATP levels were measured with an enzymatic-based kit (Fig. 5h). Taken together, these findings support the notion of perturbed mitochondrial function due to PM exposure in hOM cultures and most apparently so in PM_10–2.5_ and PM_2.5–1_.

### Transcriptomic analysis reveals alteration to cellular signaling in response to oxidative stress and mitochondrial dysfunction

**Fig. 6:**
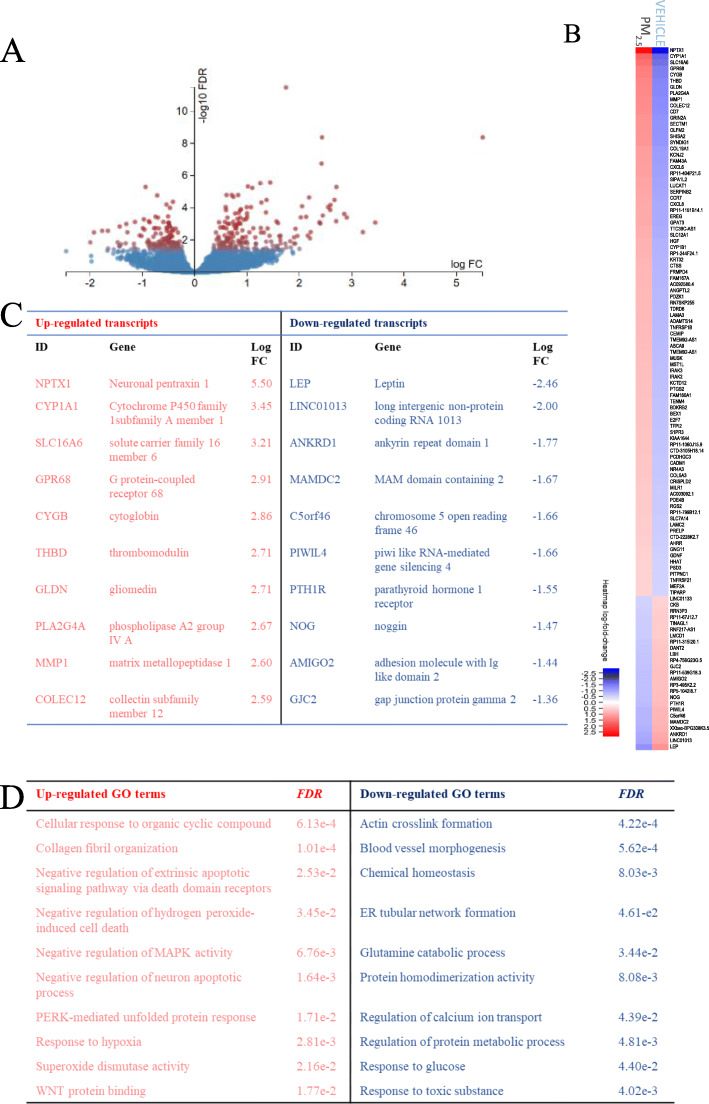
Transcriptomic analyses reveal NPTX1 as a novel target of PM in hOM cells. **a** Volcano plot of DEGs in the transcriptome of hOM cells exposed to PM_2.5–1_ reveals 377 DEGs, of which 114 are altered by two-fold or more. **b** Heatmap comparing 114 DEGs in 4 cell lines above the FDR cut-off of 0.05 and log fold change ≥1 (≥2x). **c** List of most up- and down-regulated transcripts in cells exposed to PM_2.5–1_**d** GO terms enriched with relation to significantly up- and down-regulated transcripts. N = four donors/group

To dissect PM-induced mitochondrial defects in depth and to identify potential biomarkers for PM effects in the human olfactory mucosa, PM_2.5–1_-exposed hOM cultures were subjected to RNAseq and quality control was performed on the processed data (Fig. 6a). To enrich for DEGs, the FDR was cut off at 0.05 and log fold change at 1 (< 1.5x transcript level). This yielded 114 DEGs in cells exposed to PM_2.5–1_, of which 25 were down-regulated and 89 were up-regulated (Fig. 6b). The 20 most altered genes consisted primarily of genes associated with extracellular matrix organization and cellular response to organic compounds (Fig. 6c). The most notably altered gene in the dataset encodes mitochondria-targeted neuronal pentraxin-1 (*NPTX1*), which is known to regulate BAX and mitochondrial dynamics in neurons [24]. Lastly, gene ontology (GO) terms enriched in upregulated genes showed altered pathways associated with the oxidative stress response, which agrees with our findings shown in Figs. 4 and 5 (Fig. 6d). Most notably, *NPTX1* is the most upregulated, with association to processes including cellular excitotoxicity and apoptosis. Conversely, GO terms enriched in down-regulated genes include pathways associated with homeostatic processes including cytoskeletal organization and metabolic activity.

### NPTX1 is a novel, potential biomarker for PM effects in hOM cultures

**Fig. 7:**
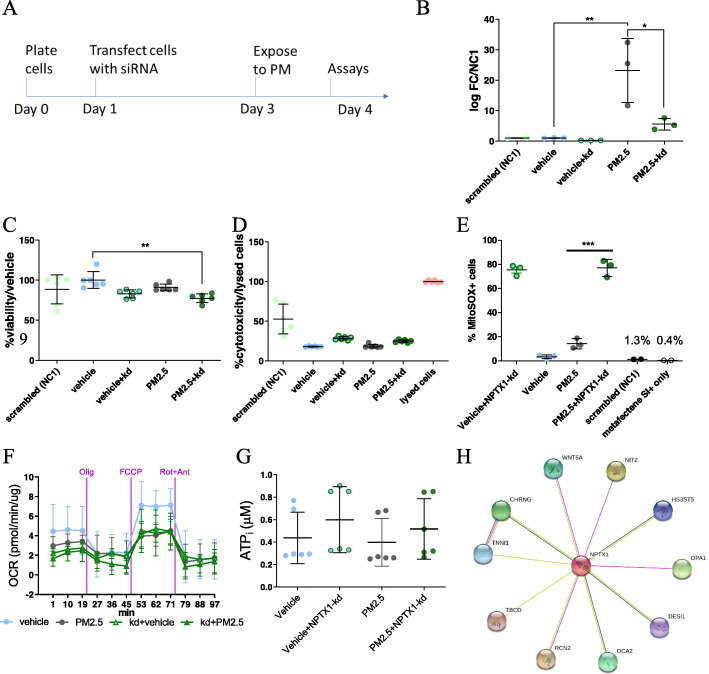
*NPTX1*-knockdown did not ameliorate mitochondrial function via ROS signalling. **a** Experimental timeline. **b** Validation of *NPTX1* expression shows a significant increase in hOM cells exposed to PM_2.5–1_ and significant suppression of *NPTX1* following RNAi in a representative cell line. *NPTX1* transcripts levels are presented as log fold change (logFC) and normalized by the reference gene, GAPDH and to the scrambled control (NC1). **c** MTT assay results of 24 h exposure to PM after 48 h treatment with siRNA. **d** LDH release assay results of 24 h exposure to PM after 48 h treatment with siRNA. **e** Proportion of mitoSOX-positive cells increased in *NPTX1*-kd cells exposed to PM. **f** Measurement of OCR with SeahorseFX technology revealed that suppression of *NPTX1* (in green lines) did not further increase the failure of cellular respiratory activity after PM exposure. **g** Intracellular ATP levels were not altered by *NPTX1*-kd. (H) STRING interaction network of NPTX1 in human. *p < 0.05 **p < 0.01 ***p < 0.0001. *N* = 3–6 wells/group

To confirm NPTX1 activation after PM exposure, qPCR results revealed a 20-fold increase of *NPTX1* transcripts, following exposure to PM_2.5–1_ (Fig. 7b). To further clarify the role of *NPTX1* upon PM exposure in hOM cultures, we next applied a siRNA approach targeting *NPTX1* transcripts (*NPTX1*-kd) in the cells according to the timeline in Fig. 7a. *NPTX1*-kd cells showed a 5-fold decrease of the transcript level, when compared to vehicle only (Fig. 7b).

Assessment of metabolic activity revealed a slight but insignificant difference between PM_2.5–1_ only (87.4 ± 0.6%) and *NPTX1*-kd + PM_2.5_ (78.4 ± 1.2%). However the cellular metabolism was significantly reduced when comparing *NPTX1*-kd + PM_2.5_ treated cells to the vehicle (Fig. 7c). There was no difference in the toxicity between the treatment groups as measured by the LDH release assay (Fig. 7d). Similar to Fig. 7b, the mitochondrial oxidative stress response was increased upon *NPTX1*-kd when compared to PM_2.5–1_ alone (Fig. 7e). The scrambled siRNA and cells incubated with transfection reagent alone exhibited similar responses to the vehicle, supporting the notion that the increasing ROS was due specifically to *NPTX1* knockdown. Taken together, these results indicate that NPTX1 may be critical for metabolic activity in hOM cultures since a 5-fold reduction in its levels is enough to cause increased oxidative stress and apoptosis. The down-regulation of *NPTX1* over-rides the effects of PM treatment alone, while the concomitant knock-down and PM treatment lead to reduced metabolic activity and increased mitochondrial ROS. The Seahorse mitostress assay result showed that *NPTX1*-kd cells exhibited reduced cellular respiration when compared to the vehicle (Fig. 7f). The concomitant exposure to PM_2.5–1_ further perturbed cellular respiration by a small fraction. There was no effect on ATP levels measured from the media of treated cells (7G). To further investigate the role of NPTX1 in mitochondria, a STRING interaction network was identified (https://string-db.org/network/9606.ENSP00000307549), revealing interactions with proteins associated with calcium signaling (TNNI1 & RCN2), metabolism (NIT2), and mitochondrial fusion (OPA1) (Fig. 7h).

Taken together, these results demonstrate that *NPTX1* is strongly induced by PM exposure. Suppression of *NPTX1* exacerbates PM-induced alterations in cellular metabolism and mitochondrial function, implying a potential protective role of NPTX1 in air pollutant exposure. This findings seems to agree with previous studies interrogating the role of NPTX1 in mitochondrial dysfunctions [25, 26].

## Discussion

Here we report the generation and utilization of a novel human cell model of the olfactory mucosa for analysis of air pollutant effects in a physiologically relevant context. We demonstrate that urban PM induces alterations in the cellular metabolism of hOM cells, characterized by altered mitochondrial function. We also identify a mitochondria-related, novel target of PM effects in hOM cells, NPTX1.

The olfactory mucosa, a neural tissue that consists of a mixture of diverse cell types, acts as the first line of defense against inhaled agents, including air pollutants. Here we utilized nasal biopsy-derived mucosal monolayer cultures from four healthy subjects, to determine how urban PM exposure affects its functions. These cells are found in the human olfactory lamina propria and epithelium in vivo, thus demonstrating the physiological relevance of our in vitro model. In contrast to cultures of the nasal epithelium, which are harvested by superficial nasal scraping or brushing, the hOM cultures are obtained by biopsy of the nasal septum approximately 1 cm from the roof of the nasal cavity, as reported [17, 18]. This technique ensures that the culture also contains other cell types besides epithelial cells. Unlike previously published reports, which have cultured the cells in neurosphere induction media, the current paper utilizes hOM cells cultured in growth media, since the aim is to model the human olfactory mucosa in its native, physiological state, without influencing differentiation or alterations in the proportions of various cell types.

Although previous studies have reported olfactory mucosa alterations due to PM exposure in animals [27, 28], and olfactory dysfunction is reported to occur in humans exposed to high levels of air pollution [29], according to our knowledge, this paper is the first to demonstrate how PM exposure affects cultured hOM cells. There exist a few papers on air pollution effects in other in vitro nasal models such as nasal epithelial cells, which have demonstrated inflammation [30, 31] and disruption of the epithelial barrier [14, 32] caused by diesel exhaust particles or PM_2.5–1_. Epigenetic alterations have been demonstrated in nasal epithelial cells of children exposed to PM_2.5–1_ in utero [33] and diesel exhaust particle exposure in cultures of nasal fibroblasts has been shown to induce inflammation [34]. It is important to note that it is specifically the olfactory mucosa that is connected to the olfactory bulbs, enabling the passage of inhaled agents into the brain [35]. Projections from the mucosal olfactory neurons form the olfactory nerve terminates in the olfactory bulb. While air pollution effects on the human olfactory mucosa have not been studied, several studies have reported alterations to the olfactory bulbs of animals upon exposure to various pollutants, including diesel exhaust particles and PM [35–38]. The effects of pollutants on the olfactory bulb have been largely attributed to increased inflammation [38, 39].

In line with the above-mentioned studies, our results in hOM cultures demonstrate that urban PM exposure induces an inflammatory response, likely due to the presence of inflammatory cells in the cultures. Urban PM have been reported to contain bacterial endotoxins, potentially accounting for the secretion of cytokines in primary hOM cells [40]. The inflammatory response to PM exposure was primarily characterized by increased IL-6 secretion. In the presence of an inflammatory milieu, modelled here by treatment with IFN-ɣ/TNFα, the mounted IL-6 response was further augmented by PM exposure, suggesting an additive inflammatory effect of PM treatment in the presence of an underlying inflammatory insult. A similar pattern of secretion was observed for GM-CSF, a product of stimulated epithelial cells. These results are important regarding human exposure as they suggest that individuals suffering from existing olfactory inflammation are possibly at risk of generating a heightened inflammatory response upon PM exposure. In fact, air pollution exposure has been shown to worsen symptoms in individuals with allergy-induced inflammation and bronchial asthma [41]. As has been reported by others [42], we also observed increased IL-8 secretion upon PM exposure, but this was not further augmented by existing inflammation. MCP-1, a chemokine responsible for recruiting monocytes, T cells and dendritic cells to the site of inflammation, was reduced upon PM exposure when co-treated with IFN-ɣ/TNFα. The observed reduction of MCP-1 in co-treated hOM cells could result from activation of the anti-oxidative stress response, which in turn diminishes chemokine secretion [43]. Deciphering the reasons behind the MCP-1 response requires further studies that are beyond the scope of this paper. Collectively, these findings suggest that the hOM cells can mount an inflammatory response to PM exposure that depends on PM size and the presence of underlying inflammation.

Oxidative stress and mitochondrial dysfunction has been reported to occur in diverse cell types exposed to PM [4, 42, 44–47]. Our findings of increased oxidative stress in hOM cells upon PM exposure are in line with published findings in an immortalized nasal epithelial cell line showing increased levels of ROS upon exposure to PM_2.5–1_ [48]. Given that the mitochondria are a major subcellular source of ROS, it was imperative to focus detailed mechanistic studies to this organelle responsible for cellular energetics. Our results are the first to report mitochondrial alterations in PM-exposed primary cells of the human OM. Similar to published reports in the immortalized neuroblastoma SH-SY5Y cells [49], our findings demonstrate that exposure to PM resulted in reduced metabolic activity, and decrease in mitochondrial respiration. Our results of impaired mitochondrial function due to PM exposure are corroborated by an in vivo study demonstrating PM_2.5–1_ effects in the nasal mucosa of rats [28]. Our findings, together with the published reports in various models, suggest that mitochondria are a key target of PM exposure. It is important to note that most in vitro studies, including this study, focus on short-term effects of air pollutants in order to provide mechanistic insight into harmful PM effects. Given the technical challenges of studying long-term exposure effects in cultured cells, future studies should focus on assessing air pollutant induced mitochondrial effects in long-term exposure studies of animal models.

Our transcriptomic analysis revealed that exposure to PM_2.5–1_ enriches for pathways that are commonly invoked as cellular defense mechanisms against stress [50, 51]. Notably, up-regulated pathways that are reflected in our RNAseq data include mitochondrially driven apoptosis, responses to oxidative stress and extracellular matrix organization. In addition, the highly upregulated transcript CYP1A1 has been shown to be induced by polycyclic aromatic hydrocarbons, thus the observed increase is to be expected [52]. Also, SLC16A6 and GPR68 have been shown to be increased in human macrophages upon exposure to polycyclic aromatic hydrocarbons [53]. Interestingly, cytoglobin is also a regulator of nitic oxide production, which was reported to be induced in macrophages exposed to PM [54]. The association of serum leptin to chronic exposure to air pollutants has been reported in the MOBILIZE Boston study [55]. These markers may be applicable as biomarkers of acute exposure to air pollutants from easily accessible samples such as blood. To date, we are the first to report activation of *NPTX1* and *gliomedin* in primary hOM responding to PM exposure. NPTX1 is a neuron-specific gene that targets the mitochondria to initiate the pro-apoptotic signaling cascade. Our findings are in agreement with previous reports of NPTX1 as a driver of mitochondrial dysfunction resulting from hypoxia or excitatory toxicity [25, 26, 56].

In this study, transient suppression of *NPTX1* coupled to PM exposure resulted in a massive increase in mitochondrial ROS, that was over three-fold higher than that induced by PM exposure alone. It is possible that transient transfection of the siRNA pool prior to exposure to PM may disrupt membrane integrity, resulting in increased ROS. Considering that *NPTX1* expression is increased upon exposure to PM, we speculate that it may act as a beneficial cell response to exposure. This is corroborated by the finding that NPTX1 suppression coupled to PM adversely affected cellular health. Given the fact that NPTX1 has been implicated in neurodegeneration, brain cancers and in compromised blood-brain barrier [56–58], further investigation of NPTX1 may be of interest especially since air pollution exposure is also linked to neurodegeneration and blood brain barrier dysfunction. Particular interactors of NPTX1 that are reported to mediate bioenergetics such as NIT2, OPA1, RCN2 may act together in response to cellular stress [59–61]. Nonetheless, given our initial observations, further research may enable a better understanding of this interaction which is beyond the scope of this study.

## Conclusion

In this study, we present primary hOM cells as a novel cellular model to interrogate air pollutant effects. We demonstrate that exposure to urban PM of different class sizes leads to a mild inflammatory response, increased oxidative stress and mitochondrial dysfunction. Specifically, cellular respiration and mitochondrial ROS are shown to be implicated in mitochondrial dysfunction. In addition, exposure to PM resulted in alteration to cytoplasmic and mitochondria-targeted transcripts associated with apoptosis and extracellular matrix organization which can be applied to early biomarker discovery in future air pollution studies. Further investigation of mitochondria targeted NPTX1 revealed its potential role in protection against PM exposure. Future studies may further dissect the interactome of NPTX1 in mitochondrial function, given that NPTX1 has been implicated in multiple age-related diseases associated with air pollution exposure.

## Supplementary information

**Additional file 1: Table S1.** Cell Line Information. **Table S2.** Chemical composition of PMs used in this study. **Table S3.** Taqman assays used in this study. **Figure S1.** Initial dose response assessment of PM10–2.5 and PM_2.5–1_.

## Data Availability

All data generated or analysed during this study are included in this published article and its supplementary information files. The RNAseq data during the current study is available from the corresponding author on reasonable request.

## Abbreviations

CBA: Cytometric bead array
DEG: Differential gene expression
GM: Growth medium
hOM: Human olfactory mucosa
LDH: Lactate dehydrogenase
mtDNA: Mitochondrial DNA
MTT: 3-[4,5-dimethylthiazole-2-yl]-2,5-diphenyltetrazolium bromide
NIM: Neurosphere induction medium
NM: Neuronal medium
NPTX1: Neuronal pentraxin 1
OCR: Oxygen consumption rate
PM: Particulate matter
qPCR: Quantitative polymerase chain reaction
RNAi: RNA interference
RNAseq: RNA sequencing
ROS: Reactive oxygen species
siRNA: Mall interfering RNA
