# Supplementary material for: Urban air particulate matter induces mitochondrial dysfunction in human olfactory mucosal cells
Source: Part Fibre Toxicol. 2020 Jun 1;17:18. doi: 10.1186/s12989-020-00352-4 (PMC7268298; doi:10.1186/s12989-020-00352-4)
Supplement: Supplementary file 1 — Additional file 1: Table S1. Cell Line Information. Table S2. Chemical composition of PMs used in this study. Table S3. Taqman assays used in this study. Figure S1. Initial dose response assessment of PM10–2.5 and PM2.5–1. [file 12989_2020_352_MOESM1_ESM.pdf]

Supplementary Information

Table S1. Cell Line Information

**Cell Line ID**	**Sex, Age**	**Lifestyle**
C1 100 08 0011	M, 63	Non-smoker
C1 100 08 0013	M, 54	Non-smoker
C1 100 08 0016	M, 60	Non-smoker
C1 100 15 0003	M, 68	Smoker

Table S2. Chemical composition of PMs used in this study

**Component**	**PM1-0.2**	**PM2.5-1**	**PM10-2,5**
**Total metals (µg/mg)**	20.57	29.6	61.11
**SO42- (µg/mg)**	300	260	22.6
**PO43- (µg/mg)**	1.5	3.4	5.6
**NO3- (µg/mg)**	110	130	99.1
**Total PAH (ng/mg)**	126.7	293.1	159.1
**Genotoxic PAH (ng/mg)**	80.8	207.5	118.4
**Air quality (µg/m^3)**	74.8		66.1
**Wind speed (m/s)**	2.4		
**Temperature (°C)**	27.1		
**Relative humidity (%)**	52.0		

Data modified from Jalava et al, 2015. The PMs were sampled from the approximate location, N32_07.1520, E118_56.918' between 18th and 23rd May 2013. The PM samples were collected from the Nanjing University Xianlin campus in Nanjing, China. The sampling location can be described as an urban background site. The sampling station was located on the rooftop of a 5-floor university building and during the sampling, meteorological conditions, wind directions and particulate concentrations were continuously monitored. The PM samples were collected for approximately 12 hours a day. The samples were collected with a high-volume cascade impactor that segregates the particles according to size with three different cut-off points. The sampler was equipped with a pre cut-off PM_{10} inlet. The PM samples were collected on polyurethane foam (PUF) sampling material and extracted by previously described methodology (3). Shortly, the PUF material was placed into glass tubes filled with methanol and sonicated for 30 min. This treatment was repeated and the suspension was collected into a flask. The excess methanol was evaporated with a rotary evaporator and the sample was distributed to sample tubes based on mass. The remaining methanol was evaporated under nitrogen flow to yield dry particulate mass. The samples were reconstituted by adding DMSO and water to tubes and sonicating them before dosing on the cells.

Table S3. Taqman assays used in this study

Human Gene Symbol	Taqman Assay Mix ID
ASCL1	Hs00269932_m1
GAPDH	Hs02758991_g1
ZNF232 (MT-ND4)	Hs02596876_g1
NESTIN	Hs04187831_g1
NEUROG1	Hs01029249_s1
NPTX1	Hs00982601_m1
OMP	Hs01087269_s1
PAX6	Hs01088114_m1
SOX2	Hs01053049_s1
TP63	Hs00978340_m1
TUBB3 (TUJ1)	Hs00964963_g1

Figure S1. Initial dose response assessment of $PM_{10-2.5}$ and $PM_{2.5-1}$

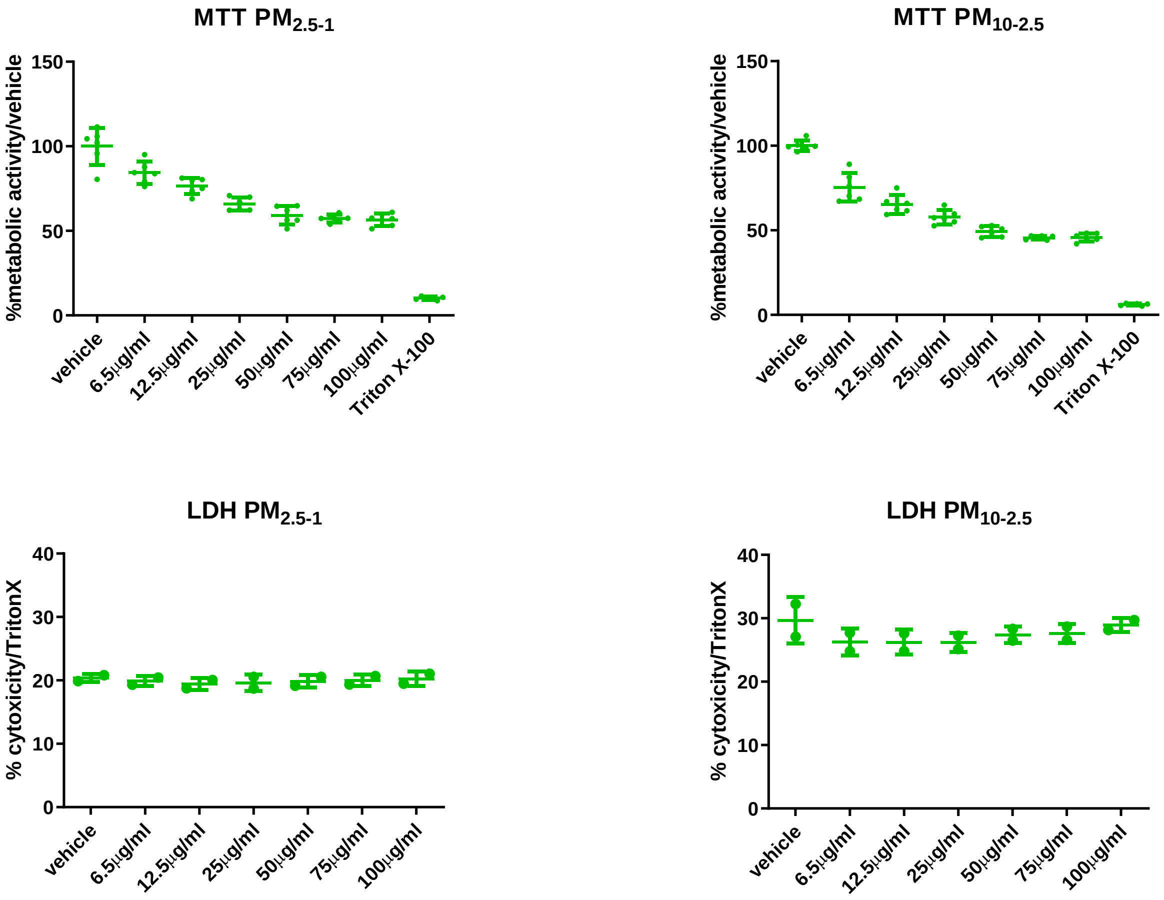

Dot plots showing metabolic activity and LDH release in response to 24h exposure to PMs in the donor cell line C1 100 08 0013.
